# Comparative outcomes of catheter-directed thrombolysis versus AngioJet pharmacomechanical catheter-directed thrombolysis for treatment of acute iliofemoral deep vein thrombosis

**DOI:** 10.1016/j.jvsv.2023.08.010

**Published:** 2023-08-23

**Authors:** Tao Kang, Yao-Liang Lu, Song Han, Xiao-Qiang Li

**Affiliations:** a Department of Vascular Surgery, The Second Affiliated Hospital of Soochow University, Suzhou, China; b Department of Vascular Surgery, The First People’s Hospital of Taicang, Taicang, China; c Drum Tower Hospital, Affiliated Hospital of Nanjing University Medical School, Nanjing, China

**Keywords:** Endovascular procedures, Percutaneous aspiration thrombectomy, Thrombolytic therapy, Treatment Outcome, Venous thrombosis

## Abstract

**Objective:**

The objective of this study was to compare the outcomes of pharmacomechanical thrombolysis and thrombectomy (PCDT) plus catheter-directed thrombolysis (CDT) vs CDT alone for the treatment of acute iliofemoral deep vein thrombosis (DVT) and summarize the clinical experience, safety outcomes, and short- and long-term efficacy.

**Methods:**

We performed a 4-year retrospective, case-control study. A total of 95 consecutive patients with acute symptomatic iliofemoral deep vein thrombosis (DVT) with a symptom duration of ≤7 days involving the iliac and/or common femoral veins underwent endovascular interventions. The patients were divided into two groups according to their clinical indications: PCDT plus CDT vs CDT alone. Statistical analyses were used to compare the clinical characteristics and outcomes between the two groups. Additionally, the patients were followed up for 3 to 36 months after treatment, and the proportions of post-thrombotic syndrome (PTS) and moderate to severe PTS were analyzed using the Kaplan-Meier survival method.

**Results:**

A total of 95 consecutive patients were analyzed in this retrospective study, of whom, 51 underwent CDT alone and 44 underwent PCDT plus CDT. Between the two groups, in terms of immediate-term efficacy and safety, significant differences were found in the catheter retention time (60.64 ± 12.04 hours vs 19.42 ± 4.04 hours; *P* < .001), dosages of urokinase required (5.82 ± 0.81 million units vs 1.80 ± 0.64 million units; *P* < .001), the detumescence rate at 24 hours postoperatively (48.46% ± 8.62% vs 76.79% ± 7.98%; *P* = .026), the descent velocity of D-dimer per day (2266.28 ± 1358.26 μg/L/D vs 3842.34 ± 2048.02 μg/L/D; *P* = .018), total hospitalization stay (6.2 ± 1.40 days vs 3.8 ± 0.70 days; *P* = .024), number of postoperative angiograms (2.4 ± 0.80 vs 1.2 ± 0.30; *P* = .042), and grade III venous patency (>95% lysis: 54.5% vs 68.6%; *P* = .047). Furthermore, during the follow-up period, significant differences were found in the incidence of PTS (Villalta scale ≥5 or a venous ulcer: 47.0% vs 27.7%; *P* = .037), and the incidence proportion of moderate to severe PTS at 12 months (15.7% vs 4.5%; *P* = .024) and 24 months (35.3% vs 11.4%; *P* = .016).

**Conclusions:**

Compared with CDT alone, in the iliofemoral DVT subgroup with a symptom duration of ≤7 days, PCDT plus CDT could significantly relieve early leg symptoms, shorten the hospitalization stay, reduce bleeding complications, promote long-term venous patency, and decrease the occurrence of PTS and the incidence proportion of moderate to severe PTS. Thus, the short- and long-term outcomes both support the superiority of PCDT plus CDT vs CDT in this subgroup.

**Article Highlights**

• **Type of Research:** A single-center retrospective case-controlled study
• **Key Findings:** Pharmacomechanical thrombolysis and thrombectomy plus catheter-directed thrombolysis (CDT) achieved higher rates of grade III venous patency, shorter hospital stays, and a decreased incidence of post-thrombotic syndrome compared with CDT alone.
• **Take Home Message:** Both short- and long-term outcomes support the superiority of pharmacomechanical thrombolysis and thrombectomy plus CDT vs CDT alone in the acute iliofemoral deep vein thrombosis subgroup with a symptom duration of ≤7 days.

Venous thromboembolism, including pulmonary embolism (PE) and deep vein thrombosis (DVT), is a common and severe health condition,^1^ with an incidence of ≤1‰ to 2‰ annually.^2^^,^^3^ Of all inpatient mortalities, 7% result from acute PE.^4^ Approximately 70% to 80% of PE cases are caused by acute DVT. It is estimated that 20% to 50% of patients with acute symptomatic proximal DVT subsequently develop PTS. Also, 5% to 10% of PTS patients develop severe PTS if treated with standard anticoagulation and compression stockings alone.^5^, ^6^, ^7^, ^8^ Additionally, after discontinuing anticoagulation for those with proximal DVT, patients experience a risk of recurrence >20% during a 10-year period.^9^

The ninth edition of the CHEST guidelines^10^ recommends anticoagulant therapy alone over interventional therapy (ie, thrombolytic, mechanical, pharmacomechanical). However, in recent decades of clinical practice, thrombolytic therapy, including catheter-directed thrombolysis (CDT)^11^^,^^12^ and pharmacomechanical thrombolysis and thrombectomy (PCDT),^13^^,^^14^ has gradually occupied the dominant position in treating acute proximal DVT. The thrombolytic efficacy of CDT has been confirmed, justifying its use as first-line clinical therapy for patients with clinically symptomatic iliofemoral DVT.^12^^,^^15^^,^^16^ PCDT has been demonstrated to be a more effective and safe therapy, enhancing thrombectomy efficacy when experienced medical staff and hospital resources are available.^17^ The randomized controlled study, the PEARL (peripheral use of AngioJet rheolytic thrombectomy with a variety of catheter lengths) registry released multicenter follow-up results, indicating that for 329 AngioJet (Boston Scientific) thrombectomy cases, the grade II to III thrombus clearance rate reached 93%.^18^ However, the most extensive multicenter randomized controlled study (ATTRACT [acute venous thrombosis: thrombus removal with adjunctive catheter-directed thrombolysis] trial) previously reported that the incidence of PTS was 46.7% in the PCDT group and 48.2% in the anticoagulant alone group (*P* = .56), which demonstrated that PCDT did not prevent PTS in patients with acute proximal DVT but significantly the reduced early symptoms and PTS severity scores.^14^

Therefore, it is essential to investigate which thrombotic subgroups would benefit the most from PCDT plus CDT. Therefore, in the present study, we mainly evaluated the merits and demerits of PCDT plus CDT for iliofemoral DVT subgroups, allowing for a greater understanding of the optimal treatment course for these patients in the future.

## Methods

### Study design and study population

The study followed the ethical standards of the institutional review board and was approved by the medical ethics committee of The First People’s Hospital of Taicang (approval no. 2021-KY-251). Because of the retrospective, case-control study design, written informed consent from the patients was not required. From August 2017 to August 2021, 95 consecutive patients with acute symptomatic iliofemoral DVT with a symptom duration of ≤7 days were admitted to vascular surgery at The First People’s Hospital of Taicang. The HAS-BLED (hypertension, abnormal renal or liver function, stroke, bleeding history or predisposition, labile international normalized ratio, elderly, drugs and/or alcohol concomitantly) score was used to assess the patient's bleeding risk (Supplementary Table, online only),^19^^,^^20^ scored on a 1-point scale, and summed to a total score (range, 0-9). Patients were categorized as having a low risk of bleeding (score, 0-1 point) and were included in the CDT-alone group or a moderate risk of bleeding (score, 2-3 points), who were included in the PCDT plus CDT group. A severe risk of bleeding (score, 4-9 points) was considered an exclusion criterion. The patients' clinical records were reviewed for the demographic characteristics, clinical disease characteristics, pathogenic factors, accompanying primary diseases, and initial venous patency scores, which were analyzed (Table I). The case selection and grouping methods were chosen from studies reported in the literature using the HAS-BLED score for DVT or PE patients.^21^^,^^22^

**Table I:** Clinical baseline characteristics stratified by treatment group

Baseline characteristic	CDT group (n = 51)	PCDT group (n = 44)	*P* value
Gender			
Male	22 (43.1)	17 (38.6)	.526
Female	29 (56.9)	27 (61.4)	.668
Age, years	63.42 ± 16.20	66.69 ± 12.92	.327
Body mass index, kg/m^2^	28 (22-36)	27 (24-34)	.268
Thrombosis type			
Iliofemoral DVT	18 (35.3)	15 (34.1)	.442
Full-limb mixed DVT (iliac vein to calf vein)	33 (64.7)	29 (65.9)	.860
Predisposing factors			
No obvious factor	10 (19.6)	3 (6.8)	.146
Surgery/trauma	15 (29.4)	16 (36.4)	.582
May-Thurner syndrome	21 (41.2)	23 (52.3)	.714
Hypercoagulable state for tumor or immune disease	5 (9.8)	2 (4.5)	.682
Symptom duration, hours	108.6 ± 20.2	99.2 ± 25.6	.424
Diameter difference before treatment, cm			
Above knee 15 cm	5.62 ± 1.84	5.02 ± 2.46	.148
Below knee 15 cm	4.96 ± 1.75	4.03 ± 0.98	.244
D-dimer on admission, μg/L	13,218.26 ± 7468.62	12,846.00 ± 8462.78	.867
Original HAS-BLED score	0.59 ± 0.50	2.24 ± 0.43	**.064**
Initial score of venous patency	11.42 ± 1.96	10.88 ± 1.64	.586

*CDT,* Catheter-directed thrombolysis; *DVT,* deep vein thrombosis; *HAS-BLED,* hypertension, abnormal renal or liver function, stroke, bleeding history or predisposition, labile international normalized ratio, elderly, drugs and/or alcohol concomitantly; *PCDT,* pharmacomechanical thrombolysis and thrombectomy.

Data presented as number (%), median (interquartile range), or mean ± standard deviation.

Boldface *P* values represent statistical significance.

### Case inclusion and exclusion criteria

The inclusion criteria were as follows: (1) symptom duration of ≤7 days; (2) age ≤80 years; (3) involvement of iliac and common femoral veins (with or without involvement of other veins); (4) DVT confirmed by computed tomography venography (CTV) and adjunctive Doppler ultrasound; (5) no serious anticoagulation or thrombolysis contraindications (HAS-BLED score, 1-3 points); (6) life expectancy ≥1 year; and (7) no history of DVT or PE.

The exclusion criteria were as follows: (1) symptom duration >7 days; (2) femoral or popliteal or calf DVT; (3) severe anticoagulation and/or thrombolysis contraindications, including active bleeding, a history of severe trauma or major surgery in the previous 4 weeks, gastrointestinal bleeding or cerebral hemorrhage in the previous 3 months, uncontrollable hypertension (systolic blood pressure >180 mm Hg or diastolic blood pressure >110 mm Hg)^23^, ^24^, ^25^; (4) a severe risk of bleeding (HAS-BLED score, 4-9 points); (5) severe renal insufficiency (creatinine clearance [CrCl], <30 mL/min) or hypersensitivity to the contrast agent; and/or (6) life expectancy <1 year.

### Procedures

Vascular surgeons performed all the procedures in a standard radiology suite. To prevent iatrogenic PE during endovascular treatment,^26^^,^^27^ a temporary inferior vena cava filter was implanted in patients with a high risk of PE, as judged by the revised Geneva score,^28^ and removed after thrombus clearance during the present hospitalization. In the PCDT plus CDT group, the AngioJet catheter (6F Solent or 8F Zelante; Boston Scientific) was advanced through the thrombosis segment. The thrombolytic agent (100 mL of normal saline plus 200,000 U of urokinase [UK]) was sprayed evenly into the entire thrombus segment using the “pulse-spray” setting on the AngioJet. At 30 minutes after UK administration, thrombectomy was performed using the AngioJet catheter from the peripheral to central vein. The thrombolytic catheter (Unifuse; AngioDynamics) was placed in the residual thrombosis segment after repeated aspiration with a maximum time of 480 seconds. The Unifuse catheter was directly inserted into the thrombosis segment in the CDT-alone group. The length of the catheterization lesion segment is presented in Table II. In the two groups, balloon angioplasty of the stenosis lesion segment was performed before placement of a catheter to improve the efficiency of thrombolysis for some patients with severe May-Thurner syndrome (MTS).

**Table II:** Perioperative efficacy outcomes stratified by treatment group

Efficacy outcome	CDT group	PCDT group	*P* value
Catheter retention time, hours	60.64 ± 12.04	19.42 ± 4.04	**<.001**
Dosage of urokinase, million units	5.82 ± 0.81	1.80 ± 0.64	**<.001**
Diameter difference after treatment, cm			
Above knee 15 cm	1.09 ± 0.54	0.96 ± 0.42	.360
Below knee 15 cm	0.98 ± 0.24	0.83 ± 0.31	.422
Detumescence rate at 24 hours postoperatively, %			
Above knee 15 cm	48.46 ± 8.62	76.79 ± 7.98	**.026**
Below knee 15 cm	42.46 ± 4.28	82.46 ± 6.38	**.014**
D-dimer, μg/L			
Before discharge	896.03 ± 2236.99	499.11 ± 560.24	.312
Descent velocity (μg/L/D)	2266.28 ± 1358.26	3842.34 ± 2048.02	**.018**
Hospitalization, days	6.2 ± 1.40	3.8 ± 0.70	**.024**
Angiograms, No.	2.40 ± 0.80	1.2 ± 0.30	**.042**
Catheterization lesion length, cm	38.0 ± 10.6	22.0 ± 6.8	**.036**
Iliac venous stents, No.	24 (47.1)	18 (40.9)	.242
Grade of venous patency			
Grade Ⅰ, <50% lysis	2 (3.9)	4 (9.1)	.482
Grade II, 50%-95% lysis	14 (27.5)	16 (36.4)	.264
Grade III, 95%-100% lysis	24 (54.5)	35 (68.6)	**.047**
Complications			
Bradyarrhythmia	0	5	**<.001**
Hemoglobinuria	0	35	**<.001**
Renal function damage	0	0	NA
Bleeding	4	2	.406
Infection	2	0	NA
Acute recurrence (within 1 month)	0	2	NA

*CDT,* Catheter-directed thrombolysis; *NA,* not applicable; *PCDT,* pharmacomechanical thrombolysis and thrombectomy.

Data presented as mean ± standard deviation, number (%), or number.

Boldface *P* values represent statistical significance.

During postoperative hospitalization, 50,000 U/h of UK (1 million units of UK ≈ 5 mg of recombinant tissue plasminogen activator) was pumped continuously via the Unifuse catheter for all the patients. The prothrombin time, activated partial thromboplastin time, and plasma fibrinogen concentrations, and D-dimer and platelet counts were monitored every 4 hours. The UK dosage was decrease by one half when the fibrinogen concentration reached 1.5 g/L and replaced with heparinized saline when the fibrinogen concentration had decreased to 1.0 g/L. If clinical evidence of bleeding was present, such as hematuria, fresh frozen plasma and cryoprecipitate were transfused. Ascending venography was conducted every 24 hours to evaluate thrombolytic efficiency and adjust the catheter site. To distinguish iatrogenic PE due to the interventional surgical procedure from PE present at admission, computed tomography pulmonary angiography was performed for patients with clinical indications of PE after the procedure. Patients with MTS diagnosed by preoperative CTV and intraoperative angiography were treated with staged iliac vein stenting after thrombus clearance during the same hospitalization. The end point of the procedure was angiographic resolution or clinical improvement.

During the perioperative period, low-molecular-weight heparin at the standard dose (enoxaparin; 1 mg/kg actual body weight, was administered subcutaneously twice daily) was prescribed for those with normal renal function (CrCl, ≥80 mL/min) or mild renal impairment (CrCl, ≥50-79 mL/min). A dosage reduction of 50% of the standard dose was implemented for those with moderate renal impairment (CrCl ≥30-49 mL/min). This study excluded patients with severe renal insufficiency (CrCl, <30 mL/min). After discharge, rivaroxaban was prescribed for patients with normal renal function or mild renal impairment at 20 mg daily for 3 months. Those with moderate renal impairment received a dose reduction of 10 mg daily for 3 months. Rivaroxaban was discontinued for those with DVT provoked by transient or reversible risk factors but was prolonged by 10 mg daily for DVT provoked by persistent or progressive risk factors, such as a bedridden, malignant tumor, or a hypercoagulability state (Table III). All patients received venous stretch sock treatment for ≥6 months.

**Table III:** Follow-up outcomes stratified by treatment group

Follow-up outcome	CDT group	PCDT group	*P* value
Follow-up time, months	28 ± 4.8	29 ± 3.4	.587
Prolonged anticoagulation ≥3 months	22 (43.1)	18 (40.9)	.240
Incidence of PTS			
Venous ulcer	6 (11.8)	2 (4.5)	**.032**
Villalta score ≥5	18 (35.2)	8 (18.2)	**.042**
Total	24 (47.0)	10 (22.7)	**.037**
PTS incidence proportion			
At 3 months	0	0	NA
At 6 months	3 (5.9)	1 (2.3)	**.034**
At 12 months	16 (31.4)	7 (15.9)	**.049**
At 24 months	24 (47.0)	10 (22.7)	**.037**
Moderate to severe PTS incidence proportion			
At 3 months	0	0	NA
At 6 months	0	0	NA
At 12 months	8 (15.7)	2 (4.5)	**.024**
At 24 months	18 (35.3)	5 (11.4)	**.016**
CEAP classification at 24 months			
C1	15 (29.4)	16 (36.4)	.268
C2	27 (52.9)	22 (50.0)	.349
C3	5 (9.8)	4 (9.1)	.226
C4	4 (7.9)	2 (4.5)	.084

*CDT,* Catheter-directed thrombolysis; *CEAP,* clinical, etiologic, anatomic, pathophysiologic; *NA,* not applicable; *PCDT,* pharmacomechanical catheter-directed thrombolysis; *PTS,* post-thrombotic syndrome.

Data presented as mean ± standard deviation or number (%).

Boldface *P* values represent statistical significance.

### Assessments and follow-up

The primary study end points were short-term thrombolytic efficacy and safety. The diameter difference of the lower extremity before and after treatment and the detumescence rates (reduction in leg swelling after surgery) at 24 hours postoperatively was evaluated. The catheter retention time, UK dosages, descent velocity of D-dimer, length of hospitalization, number of ascending angiograms during hospitalization, and incidence of complications were also recorded. The venous patency scores were individually assessed on venography by two reviewers, as proposed by Porter et al^29^: the vein of the lower limb was divided into seven segments: inferior vena cava, common iliac vein, external iliac vein, common femoral vein, proximal femoral vein, distal femoral vein, and popliteal vein. Zero points were assigned for complete patency, one for partial patency, and two for complete occlusion. The venous patency rate was calculated as follows: (prethrombolytic patency score − post-thrombolytic patency score)/prethrombolytic patency score × 100%. The grade of venous patency was as follows: grade I, <50% lysis; grade II, 50% to 95% lysis; or grade III, >95% lysis. The detumescence rate was measured as follows: (limb circumference difference before intervention − limb circumference difference at 24 hours after intervention)/limb circumference difference before intervention × 100%.

The secondary study end point was the occurrence of PTS and the proportion of moderate to severe PTS incidence during the follow-up period. All the patients were followed up in the outpatient department at 1, 3, 6, 12, 24, and 36 months after the procedure. Doppler ultrasound combined with CTV was performed to assess patency and valve function. The venous patency and Villalta scores were assessed according to the outcomes and complications.^30^^,^^31^ The Villalta scale lists five symptoms (ie, cramps, itching, pins and needles, leg heaviness, pain) and six signs (ie, pretibial edema, skin induration, hyperpigmentation, venous ectasia, redness, pain during calf compression), scored on a 4-point scale (range, 0-3 points) and summed to a total score (range, 0-33 points). Patients were then categorized as having no PTS (score, 0-4 points), mild PTS (score, 5-9 points), moderate PTS (score, 10-14 points), or severe PTS (score ≥15 points or the presence of ulcer).

### Statistical analysis

Statistical analyses were performed using SPSS, version 21.0 (IBM Corp) and Prism, version 7.0 (GraphPad) software. The numerical variables were analyzed using the independent sample *t* test, expressed as Xˉ ±S. Categorical variables were analyzed using the χ^2^ test or Fisher exact probability test. Independent or paired quantitative variables, including the venous patency rate and follow-up time, were compared using the Wilcoxon signed ranks test. The incidence rate of complications was analyzed using the χ^2^ test. The incidence rate of PTS and the proportion of moderate to severe PTS between the two groups were compared using the log-rank test and analyzed using Kaplan-Meier survival curves. For all analyses, *P* < .05 was considered statistically significant.

## Results

### Clinical efficacy

A total of 12 patients were excluded because of a high risk of bleeding (score, 4-9) and underwent inferior vena cava filter implantation because of anticoagulant and thrombolytic contraindications. A total of 95 consecutive patients were enrolled. Of the 95 patients, 51 underwent CDT alone and 44 underwent PCDT plus CDT. Between the two groups, significant differences were found in the catheter retention time, UK dosage, detumescence rate, number of angiograms, descent velocity of D-dimer, length of hospitalization, and grade of postoperative venous patency. However, no significant differences were found in the postoperative diameter or number of iliac venous stents (Table II).

### Safety outcomes

During treatment, no cases of PE as assessed by clinical symptoms and postoperative computed tomography pulmonary angiography and no in-hospital mortality or other fatal complications were observed in either group. In the PCDT plus CDT group, two cases of minor access puncture site hemorrhage were observed, with no catheter-related infection. In the CDT-alone group, four cases of postoperative bleeding complications occurred, including three cases of hemorrhage at the puncture or catheter site and one case of gastrointestinal bleeding. Additionally, two cases of catheter-related infection were observed in the CDT-alone group.

In the CDT-alone group, of the two patients with a catheter-related infection, one had had a preoperative concomitant skin infection and the other had a history of an indwelling catheter for >5 days. Sensitive antibiotics were used to control the infection after removing the thrombolytic catheter, and the prognosis was satisfactory. In the PCDT plus CDT group, the indwelling catheter time was relatively shorter, and no catheter-related infection occurred. However, five cases of bradyarrhythmia occurred during AngioJet aspiration and 35 cases of asymptomatic hemoglobinuria occurred postoperatively. All these patients gradually recovered well after active treatment. Two patients experienced acute thrombotic recurrence; one occurring 1 week later and the other, 1 month later. Both patients were rehospitalized, and catheter thrombolytic therapy was performed. No acute DVT recurrence was found in the CDT-alone group. No renal function damage occurred in the two groups (Table II).

### Clinical outcomes

The patients in both groups were followed up for 3 to 36 months. The CEAP (clinical, etiologic, anatomic, pathophysiologic) classification and Villalta scores conformed to the diagnosis of PTS, showing statistically significant differences (Table III). We performed a Kaplan-Meier curve and log-rank test to analyze the incidence of PTS and the proportion of moderate to severe PTS, finding statistically significant differences. The incidence of PTS in the CDT-alone group and PCDT plus CDT group at 24 months after surgery was 47.0% and 27.7%, respectively (*P* = .037; Fig, *A*). The corresponding incidence proportion of moderate to severe PTS at 12 months was 15.7% and 4.5% (*P* = .024) and at 24 months was 35.3% and 11.4% (*P* = .016; Fig, *B*).

**Fig:**
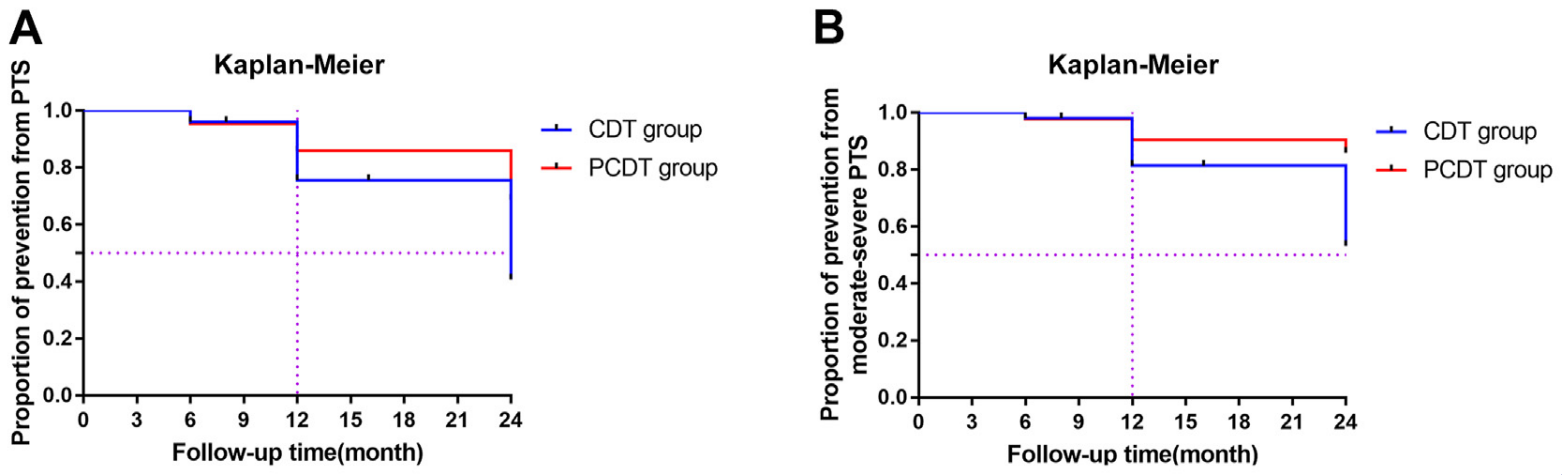
Kaplan-Meier curve analysis of post-thrombotic syndrome (PTS) stratified by treatment group. **A,** Incidence of PTS between the catheter-directed thrombolysis (CDT) group and pharmacomechanical thrombolysis and thrombectomy (PCDT) group at 24 months after surgery was 47.0% and 27.7%, respectively (*P* = .037). **B,** Incidence proportion of moderate to severe PTS in the CDT and PCDT groups at 12 and 24 months was 15.7% and 4.5% (*P* = .024) and 35.3% and 11.4% (*P* = .016), respectively, with statistically significant differences (*P* < .05).

## Discussion

Numerous clinical studies have demonstrated that PCDT is safe and effective and, when combined with early stent placement for MTS, can significantly reduce the thrombolytic time and dosage and the need for repeated angiography.^32^^,^^33^ For some elderly patients with a high risk of bleeding, PCDT has been deemed more suitable, with a more comprehensive range of indications than CDT alone.^34^^,^^35^ In the present study, 44 of the 95 patients had a higher risk of bleeding (HAS-BLED score, 2-3 points). AngioJet thrombectomy, because of its relatively shorter thrombolysis time and lower thrombolytic dosage, was deemed the optimal choice. Therefore, patients with a HAS-BLED score of 0 to 1 point were enrolled in the CDT-alone group, and those with a HAS-BLED score of 2 to 3 points were enrolled in the PCDT plus CDT group.

Several randomized controlled trials have shown the benefit of PCDT for venous patency and preventing PTS.^13^^,^^18^^,^^36^, ^37^, ^38^ However, the results of the largest trial, the ATTRACT trial, in contrast to the previously cited studies, demonstrated that PCDT did not prevent PTS, and 92 patients with DVT with a symptom duration of ≤14 days showed no differences in the occurrence of PTS at 2 years of follow-up between the PCDT group and anticoagulation-alone group. Additionally, the ATTRACT subgroup analysis of 300 patients with femoral–popliteal DVT found no differences in the development of PTS or the severity of PTS between the two groups.^39^ By contrast, another ATTRACT subgroup analysis of 391 patients with iliofemoral DVT found the severity and proportion of moderate to severe PTS were decreased in the PCDT group, suggesting that patients with acute iliofemoral DVT were more likely to benefit from PCDT.^40^ The risk of PTS is directly correlated with the onset of DVT symptoms and the freshness of the thrombosis. Therefore, the American Heart Association guidelines recommend that patients with acute iliofemoral DVT (symptoms <21 days) and a low bleeding risk are more suitable for CDT or PCDT.^41^ However, the ATTRACT trial enrolled patients with a symptom duration of ≤14 days. Similarly, Mewissen et al^42^ confirmed that grade III complete thrombolysis was achieved in 34% of patients with acute DVT (symptom duration ≤10 days) and 19% of patients with chronic DVT (symptom duration ≥10 days).

Based on this evidence, although most studies included patients with a longer duration of symptoms, in the present study, we evaluated the iliofemoral DVT subgroup with a symptom duration of ≤7 days. We found promising clinical outcomes, allowing for greater understanding of the optimal treatment time for patients with iliofemoral DVT in the future. Compared with CDT, the use of AngioJet thrombectomy resulted in statistically significant differences in the catheter retention time, UK dosage, detumescence rate, descent velocity of D-dimer, hospitalization stay, number of angiograms postoperatively, grade III venous patency, incidence of PTS, and incidence proportion of moderate to severe PTS (*P* < .05; Table II; Fig). Therefore, we surmise that PCDT plus CDT is superior to CDT alone, although higher rates of systemic complications in terms of bradyarrhythmia and hemoglobinuria were noted and warrant further investigation.

Several randomized controlled trials have reported the occurrence of complications.^43^^,^^44^ The occurrence of bleeding complications is closely associated with the dosage of UK, and the catheterization duration and repeated thrombolytic catheter angiography contribute to an increased probability of infection. The thrombolysis time of the CDT-alone and PCDT plus CDT groups was 60.64 ± 12.04 hours vs 19.42 ± 4.04 hours. The UK dosages for the CDT-alone and PCDT plus CDT groups were 5.82 ± 0.81 million units and 1.80 ± 0.64 million units, respectively, with a statistically significant difference (*P* < .001). In the CDT-alone group, two cases of catheter-related infection and four cases of bleeding complications were observed, including three cases of hemorrhage at the puncture or catheter site and one case of gastrointestinal bleeding. The latter patient had undergone endoscopic intestinal polypectomy 2 weeks before the current intervention and had been assigned to the low-risk bleeding group because of a HAS-BLED score of 1. The patient finally developed gastrointestinal bleeding on the third day of the UK injection. The timely cessation of thrombolytic therapy and infusion of fresh frozen plasma and cryoprecipitate controlled the bleeding. Thus, we realized we had underestimated the bleeding risk based on this patient's HAS-BLED score. However, two cases of bleeding complications occurred mainly at the puncture site, and no cases of infection were detected in the PCDT plus CDT group owing to the shorter catheterization times and lower UK dosages.

On the one hand, we believe that not every DVT patient is suitable for AngioJet thrombectomy, although it can reduce the incidence of severe bleeding and catheter-related infections compared with CDT alone. On the other hand, however, it can cause complications, including bradyarrhythmia, hemoglobinuria, and renal impairment. In the PCDT plus CDT group, the five cases of bradyarrhythmia that occurred during AngioJet aspiration were somewhat unexpected. Bradyarrhythmia is associated with activation of the vagal afferent pathways and the A1 receptor, leading to transient bradycardia and hypotension.^45^^,^^46^ In addition, 35 cases of postoperative asymptomatic hemoglobinuria occurred. The macroscopic hematuria gradually disappeared by postoperative active rehydration of diuretic and alkalized urine. No acute kidney injury due to acute tubular necrosis occurred. The mechanism of hemoglobinuria is mainly because the AngioJet system uses high-velocity saline jets to fragment and aspirate the thrombus, leading to intravascular hemolysis and hemoglobinuria. Therefore, AngioJet thrombectomy should be used cautiously in elderly patients with cardiac, hepatic, and/or renal insufficiency.^43^^,^^47^ Because of this, the ATTRACT trial proposed that patients aged >65 years were less likely to benefit from AngioJet thrombectomy than were younger patients; thus, more detailed subgroup studies, including age groups, will be our further research project.

In addition, short-term venous thrombosis recurrence was also of significant concern. In the PCDT plus CDT group, two patients experienced short-term recurrence after discharge, with no statistically significant differences between the two groups. However, we investigated the causes, including incomplete thrombus clearance due to partial aging thrombosis, too low placement of a stent to fully cover the iliac venous stenosis lesion segment, and patient characteristics, such as age, poor compliance, or irregular use of anticoagulant drugs. Therefore, the timely removal of the thrombus to ensure an unobstructed inflow tract, opening of the iliac vein lesion segment to provide an open outflow tract, and long-term standardized anticoagulation therapy are keys to effectively preventing thrombus recurrence and PTS.

Patency of the iliofemoral venous outflow after the intervention was an independent risk factor for PTS and thrombus recurrence. Ming et al^48^ reported that the incidence of PTS in patients with or without stent implantation at 12 months of follow-up was 8.0% and 37.1%, respectively. A direct or staged iliac vein stent implantation strategy will also affect the long-term patency rate of the vein. Liu et al^49^ reported that iliac vein stent implantation could effectively shorten the length of the hospital stay and improve the long-term patency rate after 1 year (97.8% vs 93.5%). In the present study, the PCDT plus CDT group had a lower incidence of PTS (27.7%) than that in the ATTRACT trial (48.2%). The main reason lies in the complete thrombus clearance allowed by the age of the DVT (symptom duration ≤7 days) and the timely opening of iliofemoral venous outflow obstruction. A total of 42 iliac venous stents were implanted using a staged strategy among the two groups, contributing to our low incidence of PTS and thrombus recurrence.

### Study limitations

Several limitations are salient in our study. First, this study has not detected differences in outcomes based on age, sex, or body mass index owing to the single-center retrospective case-control study design, small sample size, and short follow-up period. Second, China faces the same challenges with long-term medication and follow-up as other countries, and the solution to these challenges requires the joint efforts of the state, hospitals, and public on health education and management. Third, our treatment options were determined by a comprehensive assessment of bleeding risk; thus, the results could be susceptible to case selection bias, although they would not affect the experimental results significantly. Additionally, we could not add new insights regarding debatable decisions concerning the age of DVT, types and dosages of the thrombolytic agent used, or choice of thrombectomy device, but our study does represent the daily practice of our primary hospital. Finally, a multicenter, randomized controlled trial will be necessary to test and contrast the safety and efficacy of CDT and PCDT for treating iliofemoral DVT.

## Conclusions

PCDT plus CDT is feasible, safe, and effective in treating the iliofemoral DVT subgroup with a symptom duration of ≤7 days. In addition, PCDT could relieve early leg symptoms, shorten the hospitalization stay, reduce bleeding complications in the short term, increase the grade of venous patency, and decrease the occurrence of PTS and incidence proportion of moderate to severe PTS in the long term. Thus, the short- and long-term outcomes both support the superiority of PCDT plus CDT vs CDT alone for the iliofemoral DVT subgroup, although the best indications require further exploration.

## Author Contributions

Conception and design: TK, YL, XL

Analysis and interpretation: TK, SH, XL

Data collection: TK, YL, SH, XL

Writing the article: TK, YL, SH, XL

Critical revision of the article: TK, YL, SH, XL

Final approval of the article: TK, YL, SH, XL

Statistical analysis: TK, XL

Obtained funding: TK

Overall responsibility: XL

## Disclosures

None.

## Figures and Tables

**Supplementary Table (online only):** HAS-BLED (hypertension, abnormal renal or liver function, stroke, bleeding history or predisposition, labile international normalized ratio, elderly, drugs and/or alcohol concomitantly) score for bleeding risk prediction for case selection

Component	Description	Point
H	Uncontrolled hypertension; SBP >160 mm Hg	1
A	Abnormal renal and/or hepatic function; dialysis, transplant, serum creatinine >200 μmol/L, cirrhosis, bilirubin >2 × ULN, AST, ALT, and ALP >3 × ULN	1 each
S	Stroke; previous ischemic or hemorrhagic stroke	1
B	Bleeding history or predisposition; previous major hemorrhage or anemia or severe thrombocytopenia (platelet count <50,000/mm^3^)	1
L	Labile INR; TTR <60% in patients receiving VKA	1
E	Elderly; aged >65 years or extreme frailty	1
D	Drugs or excessive alcohol drinking; concomitant use of antiplatelet or NSAID and/or excessive alcohol per week	1 each

*ALP,* Alkaline phosphatase; *ALT,* alanine aminotransferase; *AST,* aspartate aminotransferase; *INR,* international normalized ratio; *NSAID,* nonsteroidal anti-inflammatory drug; *SBP,* systolic blood pressure; *TTR,* time in therapeutic range; *ULN,* upper limit of normal; *VKA,* vitamin K antagonist.
